# Momentum space imaging of σ orbitals for chemical analysis

**DOI:** 10.1126/sciadv.abn0819

**Published:** 2022-07-22

**Authors:** Anja Haags, Xiaosheng Yang, Larissa Egger, Dominik Brandstetter, Hans Kirschner, François C. Bocquet, Georg Koller, Alexander Gottwald, Mathias Richter, J. Michael Gottfried, Michael G. Ramsey, Peter Puschnig, Serguei Soubatch, F. Stefan Tautz

**Affiliations:** ^1^ Peter Grünberg Institut (PGI-3), Forschungszentrum Jülich, Jülich, Germany.; ^2^ Jülich Aachen Research Alliance (JARA), Fundamentals of Future Information Technology, Jülich, Germany.; ^3^ Experimentalphysik IV A, RWTH Aachen University, Aachen, Germany.; ^4^ Institut für Physik, Karl-Franzens-Universität Graz, NAWI Graz, Graz, Austria.; ^5^ Physikalisch-Technische Bundesanstalt (PTB), Berlin, Germany.; ^6^ Fachbereich Chemie, Philipps-Universität Marburg, Marburg, Germany.

## Abstract

Tracing the modifications of molecules in surface chemical reactions benefits from the possibility to image their orbitals. While delocalized frontier orbitals with π character are imaged routinely with photoemission orbital tomography, they are not always sensitive to local chemical modifications, particularly the making and breaking of bonds at the molecular periphery. For such bonds, σ orbitals would be far more revealing. Here, we show that these orbitals can indeed be imaged in a remarkably broad energy range and that the plane wave approximation, an important ingredient of photoemission orbital tomography, is also well fulfilled for these orbitals. This makes photoemission orbital tomography a unique tool for the detailed analysis of surface chemical reactions. We demonstrate this by identifying the reaction product of a dehalogenation and cyclodehydrogenation reaction.

## Teaser

Photoemission orbital tomography of σ orbitals opens the way to full orbital characterization of surface-adsorbed molecules.

## INTRODUCTION

In frontier orbital theory, orbital patterns are regarded as important for chemical empiricism—they determine molecular reactivities and reaction pathways (*1*, *2*). Orbitals are attributed as a reality of their own besides the total electron densities in which influential electronic structure theories are framed (*3*, *4*). This has spurred many successful efforts to image orbitals (*5*–*7*) despite subtleties of the single-electron orbital concept (*8*, *9*). Orbital imaging has also been applied to the investigation of chemical bonding (*10*–*12*). The imaging of orbital-derived local densities of states with low-temperature scanning tunneling microscopy (STM) (*6*, *10*, *11*) is particularly attractive because it combines spectroscopic energy resolution with sub-angstrom spatial resolution, and the sensitivity to orbital patterns can be improved by functionalizing the STM tip (*6*, *13*). Furthermore, time resolution in STM-based orbital imaging has been achieved (*14*).

There is, however, a fundamental drawback of STM-based orbital imaging. The method is restricted to orbitals close to the chemical potential [Fermi energy (*E*_F_)], typically in an energy range *E*_F_ ± 2 eV. In many cases, however, it would be advantageous to be able to identify, analyze, and image deeper-lying orbitals. A case in point are σ orbitals. Because their electron density is concentrated along the atom-atom interconnections in molecules, they play an important role in the local bonding between neighboring atoms. The capability to measure σ orbitals as fingerprints of local chemical structures is therefore critical.

Photoemission orbital tomography (POT) (*7*) is a recent technique for the orbital analysis and orbital imaging of molecules on surfaces. For example, valence spectra have been deconvolved model-free into orbital projected densities of states (pDOS) (*15*, *16*), orbitals have been reconstructed from experimental data in two dimensions (2D) and 3D (*17*–*19*), and orbital patterns in momentum space have been recorded with femtosecond time resolution (*20*). While it has long been recognized that angle-resolved photoelectron spectroscopy (ARPES) can image orbitals in the molecular frame, the alignment of molecules in the gas phase is a challenge (*21*–*23*). In contrast, in POT, photoelectrons are collected in the half-space above the sample surface on which the adsorbed molecules are fixed in space (*7*, *17*–*19*). The measured angular distribution of photoelectrons is governed by the spatial distribution of electrons in the initial state—the orbital. There is overwhelming evidence that, for π orbitals in conjugated molecules, this relation is a straightforward Fourier transform (*24*, *25*). This is valid if the final state of the photoelectron after photoemission can be represented by a plane wave (PW) [so-called plane wave approximation (PWA)].

Because it is based on ARPES, POT per se is not limited to a particular binding energy range if the photon energy is large enough. However, there is an important caveat. So far, the PWA has only been confirmed experimentally for π orbitals close to *E*_F_. For π-conjugated organic molecules, these originate from linear combinations of only 2p*_z_-*orbitals on identical (all carbon) atoms. Under these circumstances, the PWA is equivalent to the independent atomic center (IAC) approximation (*7*, *25*) in which the photoelectron wave function is described in terms of unbound solutions of the atomic Schrödinger equation with energy *E*_kin_ summed over all atoms of the molecule and taken in the asymptotic limit for infinite distances of the detector from the photoemitter (see Materials and Methods for details) (*26*). However, this equivalence relies on photoemission from only one type of atomic orbital. If, on the other hand, the photoemission occurs from several different atomic orbital types, for example, the carbon 2s, 2p*_x_*, and 2p*_y_-*orbitals of which σ orbitals consist, then the PWA may become less accurate because the contributions of the different atomic orbitals do not any more assemble to the simple PW final state that essentially Fourier transforms the initial state orbital. Therefore, the applicability of the PWA to the final state of the photoemission needs to be questioned in this case.

This conceptual issue of POT for σ orbitals is compounded by an experimental one. Because of the typically larger wave vectors of (the topmost) σ orbitals, larger photon energies must be chosen, such that the characteristic emission patterns of these orbitals are still included in the photoelectron horizon. However, at the higher wave vectors and higher binding energies of the σ orbitals, the intensity of substrate emissions is large, possibly causing a difficulty to distinguish orbital tomograms of the σ orbitals from the background.

Here, we address by experiments the question of whether the angular distributions of σ orbitals can be reliably measured and explained in terms of the PWA. If this was the case, then POT could straightforwardly be extended to the identification, analysis, and imaging of σ orbitals and their chemical phenomenology. This would open a much more direct way to study chemical bonding than the commonly used x-ray photoelectron spectroscopy (XPS), which provides indirect information on local bonding by measuring the influence of these bonds on the binding energies and spectral shapes of atomic core levels. With σ-based POT, one would gain the possibility to access the bonding orbitals directly.

Our model is the on-surface dehalogenation and cyclodehydrogenation of 10,10′-dibromo-9,9′-bianthracene (DBBA). DBBA and similar molecules are often used to create carbon-based nanostructures by on-surface synthesis (*27*–*29*). The initially dehalogenated positions of the nonplanar precursor molecule DBBA not only can directly engage in an Ullmann-type C─C coupling (*30*, *31*) but also can become metalated or hydrogenated. Because the products of this reaction have an influence on the structural quality of the targeted nanostructures (*28*), the analytical discrimination between these competing reactions and products is important, and we used it as a test case for POT on σ orbitals. The size of the molecular products suppresses photoelectron scattering by atoms of the metal substrate (*32*) because many atoms of the molecule are typically out of registry with the substrate, thus safeguarding the PWA as far as possible. Our model system is therefore a promising candidate to achieve our conceptual goal of proving momentum space imaging of σ orbitals for chemical analysis and, at the same time, to address an actual scientific problem in the field of on-surface dehalogenation and cyclodehydrogenation reactions.

## RESULTS

**Fig. 1.**
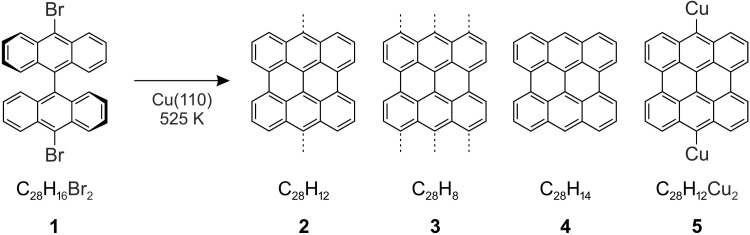
DBBA and possible reaction products on Cu(110). Dashed lines indicate bonds between carbon atoms and Cu atoms in the substrate.

**Fig. 2.**
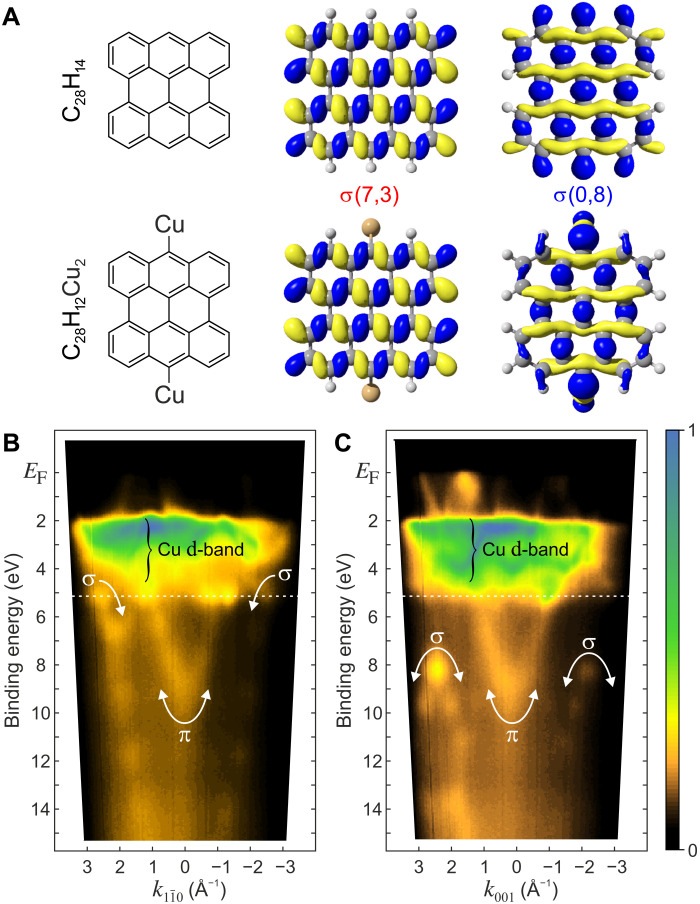
σ orbitals and ARPES band maps. (**A**) σ(7,3) and σ(0,8) orbitals of bisanthene (C_28_H_14_, **4**) (top) and metalated bisanthene (C_28_H_12_Cu_2,_ **5**) (bottom). (**B** and **C**) Band maps along the [1$\bar{1}$0] and [001] directions. π and σ bands are labeled. The white dashed lines denote binding energy *E*_b_ of the **k**_∥_ map in Fig. 4A.

**Fig. 3.**
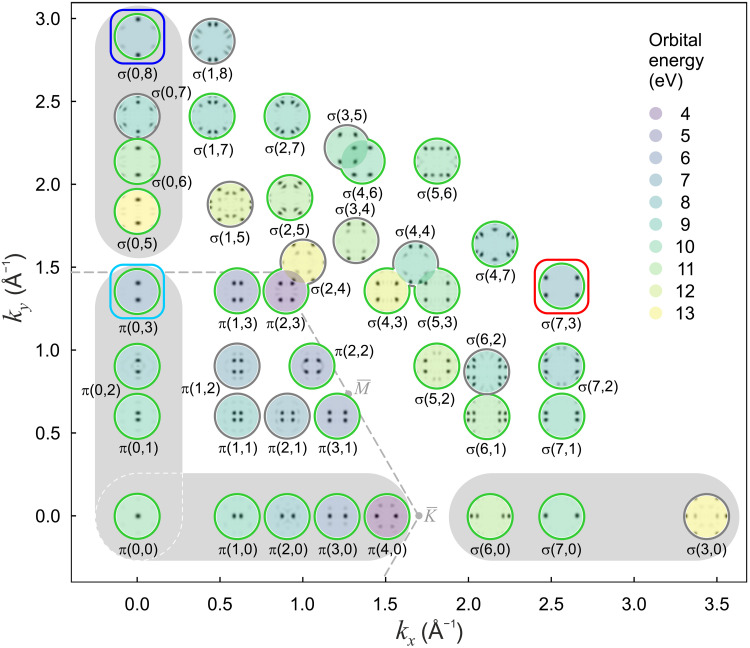
Orbitals of bisanthene (C_28_H_14_, 4). The DFT-calculated theoretical **k**_∥_ maps of the free-molecule orbitals arranged by (*k_x_* and *k_y_*) of their smallest |**k**_∥_| emission lobes. Orbital labels indicate the number of nodal planes along the two principal directions. Calculated orbital energies (zero at vacuum level) are indicated by color. Circles indicate whether these orbitals have been identified (green) or not identified (gray) in the experimental dataset. The gray-shaded areas denote orbitals of the π and σ bands marked in Fig. 2 (B and C). The dashed line marks the Brillouin zone of graphene. See fig. S2 for a plot with the real space images of the orbitals.

**Fig. 4.**
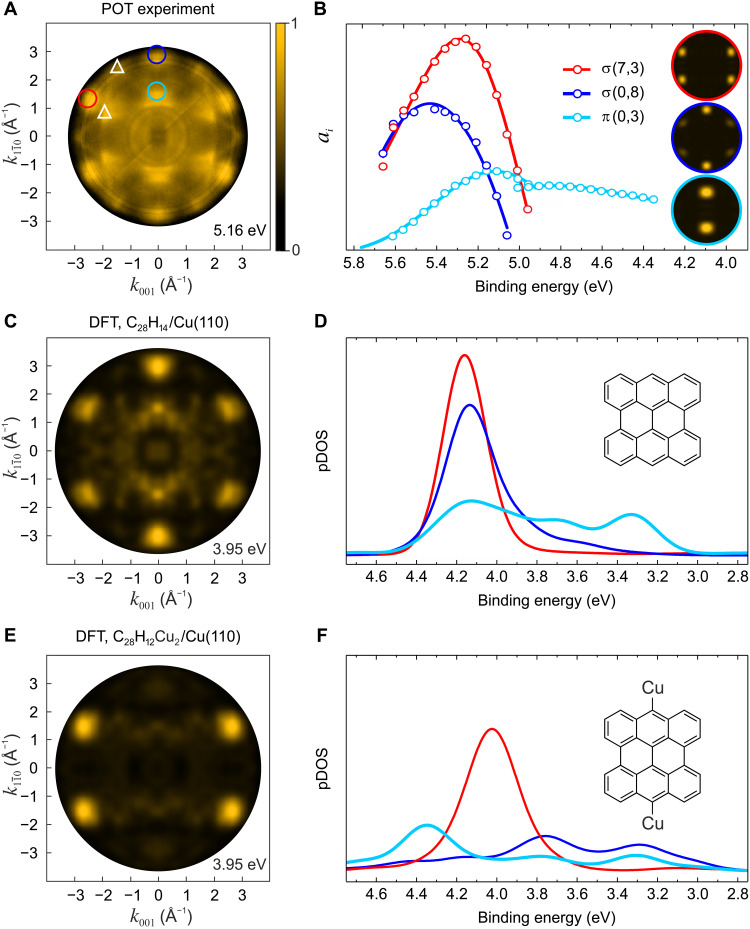
k_∥_ maps and pDOS for two possible reaction products. (**A**) Experimental **k**_∥_ map measured at binding energy *E*_b_ = 5.16 eV. The circles denote emissions from molecular orbitals [color as in (B), (D), and (F)], and the triangles denote emissions from metal states. The experimental **k**_∥_ map of clean Cu(110) is shown in fig. S8. (**B**) Experimental pDOS of σ(7,3), σ(0,8), and π(0,3) orbitals. The data points were obtained from the deconvolution of the experimental data cube *I*(*E*_b_, **k**_∥_) using the theoretical **k**_∥_ maps of free bisanthene (C_28_H_14_, **4**) (see inset). For π(0,3), two adjacent datasets with a *E*_b_ range of 0.7 eV each are displayed. (**C**) Theoretical **k**_∥_ map of C_28_H_14_/Cu(110) at the calculated *E*_b_ = 3.95 eV. (**D**) Theoretical pDOS of σ(7,3), σ(0,8), and π(0,3) orbitals of C_28_H_14_/Cu(110). (**E**) Theoretical **k**_∥_ map of C_28_H_12_Cu_2_/Cu(110) at the calculated *E*_b_ = 3.95 eV. (**F**) Theoretical pDOS of σ(7,3), σ(0,8), and π(0,3) orbitals of C_28_H_12_Cu_2_/Cu(110). (C) to (F) are based on van der Waals–corrected DFT calculations with the Perdew-Burke-Ernzerhof–generalized gradient approximation functional.

We deposited DBBA (Fig. 1, **1**) on Cu(110) (*12*, *33*). DBBA undergoes catalytic dehalogenation and cyclodehydrogenation reactions at 525 K (*12*, *33*), during which the adsorbate planarizes and the π conjugation expands to the entire fused carbon backbone, leading to the formation of a bisanthene-like species that is adsorbed flat on the surface—possible products in agreement with STM and XPS (*12*, *33*) are shown in Fig. 1. While a direct chemical bonding between the underlying substrate atoms and the carbon atoms in the 10,10′ positions (Fig. 1, **2**) (*33*), or even all carbon atoms along the zig-zag edge of bisanthene (Fig. 1, **3**), was excluded in a previous study based on the analysis of frontier π orbitals (*12*), the discrimination between bisanthene (Fig. 1, **4**) and metalated bisanthene (Fig. 1, **5**) has not yet been achieved. Several examples of metal-molecule complexes were reported in similar reactions (*28*, *31*, *34*), and hence, it is not clear how the valency of carbon atoms in question will be saturated. Both hydrogen and copper adatoms are abundantly available and mobile at elevated temperatures in the reaction environment on the surface.

In a first attempt to discern between products **4** and **5**, we computed the adsorption structures and electronic properties of both species on Cu(110) by van der Waals–corrected density functional theory (DFT). We then calculated the pDOS by projecting the eigenstates of the DFT calculation of each molecule/substrate system onto the eigenstates (orbitals) of the corresponding isolated molecule (see Materials and Methods for details). We found that neither the pDOS in the energy window between the Fermi energy and the onset of the Cu d-band nor the theoretical **k**_∥_ maps for the lowest unoccupied molecular orbital (LUMO) and highest occupied molecular orbital (HOMO), calculated by Fourier transforming the orbitals of the isolated molecules (see Materials and Methods for details), could discriminate between the two scenarios when compared to the experiment (fig. S1). Note that upon adsorption on Cu(110), the LUMO of the bisanthene-like species becomes occupied by charge transfer from the metal (*12*), making it accessible to POT, as fig. S1 proves. Crucially, Fig. 2A shows that one of the two uppermost σ orbitals is strongly affected by the replacement of the hydrogen in the 10,10′ positions by Cu adatoms, leading to the expectation that products **4** and **5** could possibly be discriminated by measuring σ orbitals.

To check whether σ orbitals are at all detectable in POT, we recorded photoemission band maps *I*(*E*_b_, *k*), i.e., photoemission intensity *I* as a function of binding energy *E*_b_ and wave vector **k**, in two azimuthal directions up to a binding energy of *E*_b_ ≃ 13 eV (Fig. 2, B and C). We know from previous work (*12*, *33*) that the bisanthene-like product of the DBBA dehalogenation and cyclodehydrogenation is orientated with its zig-zag edges along the substrate [001] direction and with the armchair edges along [1$\bar{1}$0]. In addition to the frontier π orbitals, one can clearly identify in Fig. 2 (B and C) several molecular emissions below the d-band: a π band dispersing between *E*_b_ ≃ 4 to 10 eV at |**k**_∥_| ≤ 1.5 Å^−1^ and a band between *E*_b_ ≃ 5 to 13 eV at higher |**k**_∥_|. The large |**k**_∥_| of the latter suggest that it might be of σ character.

Figure 3 displays all calculated orbitals of bisanthene in the energy range from 3 to 13.5 eV. We arranged the corresponding theoretical **k**_∥_ maps of the isolated molecule according to their smallest |**k**_∥_| emission lobes; the latter roughly scale with the number of nodal planes along *k_x_* [zig-zag direction of bisanthene and [001] direction of Cu(110)] and *k_y_* (armchair direction and [1$\bar{1}$0]). We therefore labeled the orbitals according to their type (π or σ) and the number of nodal planes (0,1,2...) along *k_x_* and *k_y_*. For instance, the HOMO π(2,3) exhibits two (three) nodal planes along *k_x_* (*k_y_*), while the σ(7,3) orbital has seven and three nodes, respectively. π(4,0) is the LUMO.

Next, we compared the theoretical **k**_∥_ maps in Fig. 3 to experimental ones generated from the photoemission intensity data cube *I*(*E*_b_, **k**_∥_) (movie S1) at fixed binding energies *E*_b_ (see Materials and Methods). This comparison is well founded for π orbitals but still to be substantiated for σ orbitals because of the uncertain applicability of the PWA for the latter. To search for individual orbitals in the experimental data cube, we used a momentum space deconvolution with theoretical **k**_∥_ maps (figs. S3 to S7) (*15*). Details of the deconvolution are explained in Materials and Methods. Of the 15 π orbitals and 27 σ orbitals in Fig. 3, we found 12 π and 18 σ orbitals in the range from *E*_b_ = 0 to 10 eV. Only nine σ orbitals were not identified with certainty. The large number of identified σ orbitals in excellent agreement with theory confirms that POT can be applied not only to p*_z_*-derived but also to s-, p*_x_*-, and p*_y_*-derived orbitals. Given the mathematical basis of the PWA (*7*, *26*) as mentioned above, this is a remarkable finding for which no theoretical explanation has been given yet. Nevertheless, the experiment speaks for itself.

## DISCUSSION

In the remainder of the paper, we focus on the two uppermost σ orbitals just below the Cu d-band, using them to discriminate between products **4** and **5** of the surface reaction in Fig. 1. In Fig. 4A, the experimental **k**_∥_ map at *E*_b_ = 5.16 eV is displayed. Its pattern is complex and shows emissions from several states: σ orbitals and π orbitals (circles), as well as high-*E*_b_ tails of the Cu d-band (triangles). The strong emission lobes at |**k**_∥_| ≥ 2 Å^−1^ can be accounted for by the two uppermost σ orbitals σ(7,3) (red) and σ(0,8) (blue), whose real space distributions are displayed in Fig. 2A. As can be discerned from their theoretical **k**_∥_ maps (Fig. 4B, inset), the emissions at **k**_∥_ = (0, ±2.89) Å^−1^ arise from σ(0,8), while those at (±2.56, ±1.39) Å^−1^ can be from both σ(0,8) and σ(7,3). To quantitatively decompose the experimental **k**_∥_ maps for varying *E*_b_, we again used the momentum space deconvolution with theoretical **k**_∥_ maps (*15*) to extract the *E*_b_-dependent contributions *a_i_* of the component orbitals to the total POT intensity distribution *I*(*E*_b_, **k**_∥_). Figure 4B shows the *a_i_*(*E*_b_) for three orbitals: σ(0,8), σ(7,3), and π(0,3). They have the meaning of an experimentally derived pDOS and can be compared to their theoretically calculated counterparts.

In Fig. 4 (C and E), we show simulated **k**_∥_ maps for bisanthene (**4**) and metalated bisanthene (**5**) on Cu(110) based on the van der Waals–corrected DFT calculation of the complete molecule/substrate system. These maps have been obtained with a damped PW as the final state, which prevents the overrepresentation of bulk states from the substrate (*35*). For bisanthene, the six bright emission lobes at |**k**_∥_| ≥ 2 Å^−1^ (Fig. 4C) correctly reproduce the experimental pattern. In contrast, for metalated bisanthene, the four emission lobes of the σ(7,3) orbital dominate, while the emission from σ(0,8) is barely visible (Fig. 4E). This already leads us to conclude that bisanthene agrees better with the experimental data, and the metalation with Cu adatoms is unlikely.

This conclusion is further corroborated by comparing the calculated pDOS of σ(0,8) and σ(7,3) in Fig. 4 (D and F) to the corresponding experimental ones in Fig. 4B. For bisanthene, the calculated pDOS for both orbitals exhibit well-defined peaks with similar intensities and energies, therein resembling closely the experiment. In contrast, the calculated σ(0,8) pDOS for metalated bisanthene is spread out broadly in energy with its maxima well separated from that of σ(7,3). This indicates that, in the van der Waals–corrected DFT calculation of the complete molecule/substrate system, the σ(0,8) orbital of metalated bisanthene hybridizes strongly with the substrate, presumably through the Cu adatoms, which are fully integrated into the lobe structure of σ(0,8) [but, notably, not of σ(7,3)] and which, upon adsorption, couple strongly to the metal surface. Because such a hybridization is not observed in the experimental pDOS of σ(0,8) (Fig. 4B), we can definitively rule out the metalated product **5**. To cross-check, we repeated the deconvolution of the experimental data cube *I*(*E*_b_, **k**_∥_) with theoretical **k**_∥_ maps of free metalated bisanthene (fig. S9). This delivers a very similar result to the experimental pDOS in Fig. 4B, which, however, is inconsistent with the calculated pDOS for the metalated species in Fig. 4F. Thus, only bisanthene as a product of the surface reaction allows a consistent interpretation of our σ orbital data. The analysis of the deep lying π(0,3) orbital is in full accord with this result (see the Supplementary Materials).

In conclusion, we advanced photoemission orbital tomography, first by substantially extending the accessible binding energy range, particularly far below the d-band of the substrate, and then by successfully detecting σ orbitals. In particular, we found that the PWA for the final state is also suited for σ orbitals. For the product of the catalytic dehalogenation and cyclodehydrogenation of DBBA on Cu(110), we demonstrated that almost the complete orbital spectrum in a wide binding energy range is detectable. Because—depending on orbital shape, adsorption site, and registry with the sample—it is usually only a small number of orbitals that provide key information regarding chemical modifications, the ability to image the complete spectrum of valence orbitals with POT in an unrivaled binding energy range provides an invaluable advantage for the analysis of surface chemical reactions. Regarding DBBA/Cu(110), we found that the product of the catalytic dehalogenation and cyclodehydrogenation reactions is bisanthene. We anticipate that the absence of a metalation with surface adatoms at the dehalogenated positions of the precursor will aid the formation of high-quality nanostructures from this surface chemical reaction.

## MATERIALS AND METHODS

### Sample preparation

Our experiments were performed in ultrahigh vacuum (~10^−10^ mbar). The Cu(110) single crystal was cleaned by several cycles of sputtering by Ar^+^ ions at 1 keV and subsequent annealing at 800 K. A film of the DBBA precursor (Sigma-Aldrich, CAS number 121848-75-7) was deposited by evaporation from a molecular evaporator (KENTAX GmbH) onto the crystal surface held at room temperature. Subsequently, the sample was annealed at 525 K to trigger the chemical reaction.

### Photoemission experiments: Band maps and k_∥_ maps

Photoemission experiments were conducted at the Metrology Light Source insertion device beamline of the Physikalisch-Technische Bundesanstalt (Berlin, Germany) (*36*). *p*-polarized ultraviolet light (photon energy = 57 eV) with an incidence angle of 40° to the surface normal was used. In this geometry, the **A**∥**k** condition (*25*), where **A** is the vector potential of the incident light and **k** is the wave vector of the photoelectrons, is approximately fulfilled for most molecular emissions in forward direction. The significance of this condition is described below. Two different types of photoemission experiments were conducted using the toroidal electron analyzer (*37*). To obtain the experimental band maps *I*(*E*_b_,$\;k_{1\bar{1}0}$) and *I*(*E*_b_*, k*_001_), the photoemission intensity was recorded in the emission angle range from −85° to +85° along the [1$\bar{1}$0] and [001] directions of Cu(110) as a function of kinetic energy *E*_kin_. The latter was converted to binding energy *E*_b_ by fitting the experimental photoemission spectra around the chemical potential by the Fermi function to define the binding energy *E*_b_ = 0 on the experimental *E*_kin_ scale. Experimental **k**_∥_ maps at a fixed binding energy *E*_b_ were obtained from the 3D data cube *I*(*E*_b_ **k**_∥_), i.e., the intensity *I* of photoemission as a function of binding energy *E*_b_ and parallel momentum **k**_∥_, recorded while rotating the sample around its normal in 1° steps. In this way, the full photoemission intensity distribution in the **k**_∥_ plane perpendicular to the sample normal was recorded.

### DFT calculations: Computational details

The geometry and electronic structure of the free and surface-adsorbed molecules were calculated in the framework of DFT. We conducted density functional calculations for both gas phase molecules and for molecules adsorbed on the Cu(110) surface. For the former, we used the quantum chemistry package NWChem (*38*), while for the latter, we used a repeated slab approach and the Vienna Ab initio Simulation Package (VASP) (*39*–*41*) program.

The results of the gas phase calculations including the molecular orbitals and the corresponding theoretical **k**_∥_ maps (see below) are available via a web-based database (*42*). In particular, the molecular orbitals of bisanthene (C_28_H_14_, **4**) and metalated bisanthene (C_28_H_12_Cu_2_, **5**) using a Perdew-Burke-Ernzerhof–generalized gradient approximation (PBE-GGA) exchange-correlation functional are accessible from this web interface using the database IDs 406 and 407, respectively. Real-space representations of bisanthene orbitals obtained with PBE-GGA with binding energies between 4 and 13 eV with respect to the vacuum level are displayed in fig. S2. In addition, we also computed the gas phase molecular orbitals of bisanthene using the range-separated hybrid functional HSE06 [ID = 480 in (*42*)] and the long-range corrected hybrid LRC-wPBEh [ID = 486 in (*42*)].

For the full molecule/Cu(110) systems, we applied the repeated slab approach. Here, the Cu(110) substrate was modeled with five atomic layers, a lattice parameter of *a* = 3.61 Å and a vacuum layer of at least 17 Å between the slabs to avoid spurious electric fields (*43*). The most favorable adsorption site for both molecules, bisanthene (C_28_H_14_, **4**) and metalated bisanthene (C_28_H_12_Cu_2_, **5**), was determined by testing several high-symmetry adsorption sites (hollow, top, short bridge, and long bridge) in a local geometry optimization approach, allowing all molecular degrees of freedom and the topmost two Cu layers to relax until forces were below 0.01 eV/Å. For these geometry optimizations, we used the GGA according to PBE (*44*) for the exchange-correlation potential with the D3 correction for van der Waals interactions (*45*). The projector augmented wave method (*41*, *46*) was used with a PW cutoff of 500 eV and a Monkhorst-Pack 3 × 3 × 1 grid of *k*-points with a first-order Methfessel-Paxton smearing of 0.2 eV. On the basis of the relaxed adsorption geometries, which were found to be the short-bridge position for bisanthene **4** and the hollow site for metalated bisanthene **5** (figs. S13 and S14), we analyzed the electronic structures in terms of the molecular orbital pDOS and simulated the photoelectron angular distribution (**k**_∥_ maps) as described in detail below. Note that, for the pDOS, we also used the range-separated hybrid functional HSE06 (*47*, *48*) for comparison (see below).

### DFT calculations: Molecular orbital pDOS

We analyzed the electronic structures of the molecule/metal interfaces by computing the molecular orbital pDOS (*35*). In addition to the full interface system with the Kohn-Sham states Ψ*_n**q**_*, we performed a DFT calculation for a free-standing layer of molecules, cut out from the full interface, leading to the molecular orbital states ϕ*_i**q**_*. We then computed the molecular orbital pDOS ρ*_i_* by projecting the Kohn-Sham orbital of the full system Ψ*_n**q**_* with Kohn-Sham energy ε*_n**q**_* onto a given molecular state ϕ*_i**q**_* and summing over the Brillouin zone and all states *n* of the full system

$$\rho_{i}(E)=\sum_{q}\sum_{n}{|\langle \phi_{iq}|\psi_{nq}\rangle |}^{2}\;\delta (E-\varepsilon_{nq})$$ (1)

In addition to the pDOS obtained from the PBE-GGA functional as shown in Fig. 4 (D and F), we also performed self-consistent DFT calculations using the range-separated hybrid functional HSE06 (*47*, *48*). The results are depicted in fig. S10 and fully support our conclusions drawn from the PBE-GGA results. Compared to the pDOS of the PBE-GGA calculation, the HSE06 functional leads to an overall shift to larger binding energies due to partial correction of self-interaction errors and thereby further improves the agreement with experiment, displayed in Fig. 4B. The pronounced difference between the two systems, C_28_H_14_/Cu(110) and C_28_H_12_Cu_2_/Cu(110), with regard to the two topmost σ orbitals, σ(7,3) and σ(0,8), remains almost identical to the PBE-GGA calculation, thereby fully supporting our conclusions.

### DFT calculations: Simulation of k_∥_ maps

Our starting point for simulating the angle-resolved photoelectron intensity was the one-step model of photoemission in which the intensity *I*(*E*_kin_, **k**_∥_) is given by Fermi’s golden rule (*49*)

$$I(E_{\text{kin}},k_{∥})\propto \sum_{i}{|\langle \Psi_{f}(E_{\text{kin}},k_{∥})|A∙p|\Psi_{i}\rangle |}^{2}\delta (E_{i}+\Phi +E_{\text{kin}}-\hbar \omega )$$ (2)

Here, **k**_∥_ = (*k_x_*, *k_y_*) is the component of the photoemitted electron’s wave vector parallel to the surface, which is related to the polar and azimuthal emission angles θ and ϕ by

$$k_{x}=|k|\;\text{sin}\theta \;\text{cos}\phi$$ (3)

$$k_{y}=|k|\;\text{sin}\theta \;\text{sin}\phi$$ (4)

where **k** is the wave vector of the emitted electron, and $E_{\text{kin}}=\frac{\hbar^{2}{|k|}^{2}}{2m}$, where ℏ is the reduced Planck constant and *m* is the electron mass. The photocurrent of Eq. 2 is given by a sum over all transitions from occupied initial states *i* described by wave functions Ψ*_i_* to the final state Ψ*_f_* characterized by the direction (θ and ϕ) and the kinetic energy of the photoemitted electron. The δ function ensures energy conservation, where Φ denotes the sample work function, *E_i_* denotes the binding energy of the initial state, and ℏω denotes the energy of the exciting photon. The transition matrix element is given in the dipole approximation, where ***p*** and **A**, respectively, denote the momentum operator of the electron and the vector potential of the exciting electromagnetic wave (*50*).

In the POT approach (*25*, *50*), the final state Ψ*_f_* is approximated by a PW. Thereby, the photocurrent *I_i_* arising from one particular initial state *i* turns out to be proportional to the Fourier transform$\;{\tilde{\Psi }}_{i}(k)$of the initial-state wave function multiplied by the polarization factor ∣**A** ∙ **k**∣^2^

$$I_{i}(k_{\|})\propto {|A∙k|}^{2}\;{|{\tilde{\Psi }}_{i}(k)|}^{2}$$ (5)

Regarding the applicability of the PW final-state approximation, it has been argued (*7*, *51*) that it can be expected to be valid if the following three conditions are fulfilled: (i) π orbital emissions from large planar molecules, (ii) an experimental geometry in which the angle between the polarization vector **A** and the direction of the emitted electron **k** is rather small (i.e., **A**∥**k** condition approximately fulfilled), and (iii) molecules consisting of many light atoms (H, C, N, and O). Experience on a variety of π-conjugated molecules adsorbed on various substrates has shown (*25*, *52*–*54*) that conditions (ii) and (iii) seem to be well satisfied within our experimental setup. Regarding the relaxation of condition (i) toward the inclusion of σ orbitals, however, only little information has been gathered so far. Here, it should be noted that the arguments leading to (i) involve a comparison of the PW final-state approximation with the more sophisticated IAC approximation. In the IAC, the final state is treated as a coherent sum of atomic-like scattering states. However, when the initial state, written as a linear combination of atomic orbitals, consists of only a single atomic orbital type, as is the case for π orbitals, it can be shown that the IAC strictly reduces to the PW final-state description, because the atomic p*_z_*-orbital can be factored out of the respective summation [see the supplementary materials of (*7*)]. In contrast, for σ orbitals, a mathematical equivalence of the IAC and PW final-state descriptions cannot be shown. This does not a priori preclude the applicability of the simple and intuitive PW approximation but asks for an experimental test.

Using the PW final-state approximation, we computed the expected (theoretical) photoemission **k**_∥_ maps *I_i_*(**k**_∥_) for an orbital *i* according to Eq. 5, both for gas phase molecules and for the full interacting systems of the molecules adsorbed on Cu(110). For gas phase orbitals, the construction of **k**_∥_ maps can also be visualized in an intuitive manner (fig. S11). Starting from the orbital in real space Ψ(*x*, *y*, *z*) (fig. S11, A and D), first, the momentum space representation of the orbital $\tilde{\Psi }$(*k_x_*, *k_y_*, *k_z_*) is computed by a 3D Fourier transform. Then, a hemispherical cut at $|k|=\sqrt{2mE_{\mathrm{kin}}/\hbar^{2}}$ (fig. S11, B and E) and multiplication with the polarization factor according to Eq. 5 lead to the **k**_∥_ map (fig. S11, C and F). As illustrated for the π(0,3) (top row in fig. S11) and σ(7,3) (bottom row in fig. S11), the main features in σ orbitals are typically located at somewhat larger **k**_∥_ values than the ones of π orbitals, and, at the same time, the larger kinetic energies lead to a reduced photoemission intensity.

Photoemission **k**_∥_ maps were also simulated for molecules adsorbed on Cu(110). Here, the starting point was Eq. 2, in which the initial states Ψ*_i_* were taken as the Kohn-Sham orbitals of the interacting molecule/Cu(110) system, and the final state Ψ*_f_* was again approximated by a PW that was, however, modified by an exponential damping factor with a damping constant of γ = 0.5 Å^−1^ to mimic the finite mean free path of the photoemitted electrons. To achieve an acceptable momentum resolution in the resulting **k**_∥_ maps, the sampling of the Brillouin zone was increased to a *k*-grid of 5 × 5 × 3. Further details about this approach have already been published elsewhere (*35*).

### Deconvolution of the experimental data cube

In the orbital deconvolution procedure (*15*, *50*), we made use of the energy and momentum dependence of photoemission to deconvolve experimental data into individual orbital contributions. This provides an orbital-by-orbital characterization of the experimentally determined density states, i.e., an experimental pDOS that can be readily compared to a computed pDOS.

We obtained the experimental pDOS by a least squares minimization procedure. The experimental photoemission data can be viewed as a data cube *I*_exp_(*E*_b_, **k**_∥_) = *I*_exp_(*E*_b_, *k_x_*, *k_y_*): the photoemission intensity *I*_exp_ measured as a function of the two momentum components parallel to the surface, *k_x_* and *k_y_*, and the binding energy *E*_b_ (corresponding to the kinetic energy *E*_kin_ as described above). The relation between *k_x_* and *k_y_* on one hand and emission angles (θ and ϕ) of the photoelectrons on the other follows from Eqs. 3 and 4. The deconvolution of the experimental data cube then consists in minimizing the squared differences between the experimental and theoretical **k**_∥_ maps

$$\chi^{2}(a_{1},a_{2},\ldots ,a_{n})=\int dk_{∥}\;{\left[I_{\text{exp}}\;(E_{b},k_{∥})-\sum_{i}^{n}a_{i}\;(E_{b})I_{i}(k_{∥})\right]}^{2}$$ (6)

by adjusting the *n* weights *a_i_*(*E*_b_) of all orbitals *i* with the theoretical **k**_∥_ maps *I_i_*(**k**_∥_) that are allowed to contribute to the measurement data. Because the minimization is performed for each binding energy *E*_b_ separately, one thereby obtains an orbital pDOS given by the weight functions *a_i_*(*E*_b_.).

From Eq. 6, it is clear that the so-obtained experimental pDOS will also depend on the set of theoretical **k**_∥_ maps *I_i_*(**k**_∥_) that are used in the deconvolution procedure. It is therefore important to check how sensitive these theoretical **k**_∥_ maps are with respect to the choice of the exchange-correlation functional. Fortunately, it turned out that these **k**_∥_ maps are robust and remain almost unaffected by the particular choice for the exchange-correlation functional. This is illustrated in fig. S12 by computing the overlaps of bisanthene orbitals generated with the PBE-GGA functional [ID = 406 in (*42*)] with those obtained from the range-separated hybrid functional HSE06 [ID = 480 in (*42*)]. More precisely, fig. S12 shows the quantity

$$|\langle \Psi_{i}^{\text{PBE}-\text{GGA}}|\Psi_{j}^{\text{HSE}}\rangle -\delta_{\text{ij}}|$$ (7)

in a gray-scale density map, where the darkest values indicate a maximum deviation of less than 9% from perfect overlap. It should be noted that, for most of the orbitals, the similarity of the PBE-GGA and HSE06 orbitals is even more pronounced and that the resulting theoretical **k**_∥_ maps from these two functionals turn out to be virtually indistinguishable. We also note that a similar finding was made when comparing the PBE-GGA orbitals with those of the long-range corrected hybrid LRC-wPBEh [ID = 486 in (*42*)] or the global hybrid PBE0 [ID = 482 in (*42*)]. We are thus confident that the experimental orbital pDOS obtained from the photoemission data cube is indeed a meaningful and robust quantity.

## Supplementary Materials

### This PDF file includes:

On-surface synthesis pDOS of the π(0,3) orbital Figs. S1 to S14

## Notes

View/request a protocol for this paper from *Bio-protocol*.
