# Supplementary material for: Momentum space imaging of σ orbitals for chemical analysis
Source: Sci Adv. 2022 Jul 22;8(29):eabn0819. doi: 10.1126/sciadv.abn0819 (PMC9307240; doi:10.1126/sciadv.abn0819)
Supplement: Supplementary file 1 — On-surface synthesis pDOS of the π(0,3) orbital Figs. S1 to S14 [file sciadv.abn0819_sm.pdf]

Science Advances
AAAS

Supplementary Materials for

Momentum space imaging of σ orbitals for chemical analysis

Anja Haags *et al.*

Corresponding author: Peter Puschnig, peter.puschnig@uni-graz.at; Serguei Soubatch, s.subach@fz-juelich.de

Sci. Adv. **8**, eabn0819 (2022)
DOI: 10.1126/sciadv.abn0819

The PDF file includes:

On-surface synthesis
pDOS of the π(0,3) orbital
Figs. S1 to S14
Legend for movie S1

Other Supplementary Material for this manuscript includes the following:

Movie S1

On-surface synthesis

The thermally induced catalytic reaction of 10,10′-dibromo-9,9′-bianthracene (DBBA) on Cu(110) results in the formation of a bisanthene-like species (often referred to as *nanographene*) and is well documented. When deposited on Cu(110) at room temperature, DBBA adopts a non-planar adsorption configuration, as revealed by a near edge x-ray absorption fine structure (NEXAFS) and scanning tunneling microscopy (STM) study (*33*). This was also confirmed by density functional theory (DFT) calculations (*12,33*).

Post-deposition annealing triggers the dehalogenation and cyclodehydrogenation of DBBA, leading to the flat bisanthene-like species that adsorbs parallel to the surface and that is studied in this paper. The reaction itself and the adsorption configuration of this molecule was confirmed by temperature-dependent x-ray photoelectron spectroscopy (XPS), NEXAFS and STM (*33*), as well as by our complementary study using the normal incidence x-ray standing wave (NIXSW) technique, which revealed a similar adsorption height (≈ 2.25 Å above the (220) Bragg plane of Cu(110)) for chemically different carbon atoms in the backbone of the reaction product.

The stoichiometry of the bisanthene-like species is confirmed by XPS, as far as the ratio of carbon atoms in different chemical environments is concerned (*12,33*). Contradicting assignments of the C 1s core level components in (*12,33*) were attributed to different interpretations of the chemical environment at the molecular periphery (deprotonated vs. hydrogenated carbon atoms along the zig-zag edge), cf. discussion in (*12*, Supplementary Note 4). Studying the valence band by photoemission revealed an occupied molecular state in the vicinity of the Fermi energy E_F for which photoemission orbital tomography (POT) clearly revealed a $\mathbf{k}_{\|}$-map that is characteristic for the lowest unoccupied molecular orbital (LUMO) of free bisanthene (*12*). This points at the charge transfer from the metal to the adsorbate, which has likewise been confirmed by DFT (*12*).

pDOS of the π(0,3) orbital

The experimental $\mathbf{k}_{\|}$-map and projected density of states (pDOS) in Fig. 4**A**,**B** also exhibit contributions of the π(0,3) orbital (light blue circles/light blue curve). Unlike the two σ-orbitals σ(7,3) and σ(0,8), π(0,3) is strongly spread out in energy. In fact, the calculated π(0,3) pDOS of bisanthene on Cu(110) resembles the experimental result much better than metalated bisanthene does, providing yet another piece of evidence in favor of bisanthene as a product of the surface reaction. Incidentally, the strong hybridization of π(0,3) reveals an intriguing insight into the chemistry at the interface between bisanthene and Cu(110): the lobes of π(0,3), located on the zig-zag chains of bisanthene, perfectly match the periodicity of the substrate lattice in the $[1\bar{1}0]$ direction and bridge three Cu atomic rows in the [001] direction (Fig. S13). This assists the hybridization of the π(0,3) orbital with the Cu(110) surface, leading to its strong energy-broadening in Fig. 4**B**. Because metalated bisanthene occupies another adsorption site (Fig. S14), its π(0,3) orbital cannot hybridize with the substrate in the same way, resulting in a different pDOS with the leading feature at larger binding energies than σ(0,8) and σ(7,3) (Fig. 4**F**). The analysis of the deep-lying π(0,3) orbital is thus in full accord with the results from the uppermost σ-orbitals and confirms the viability of σ-orbital POT for chemical analysis.

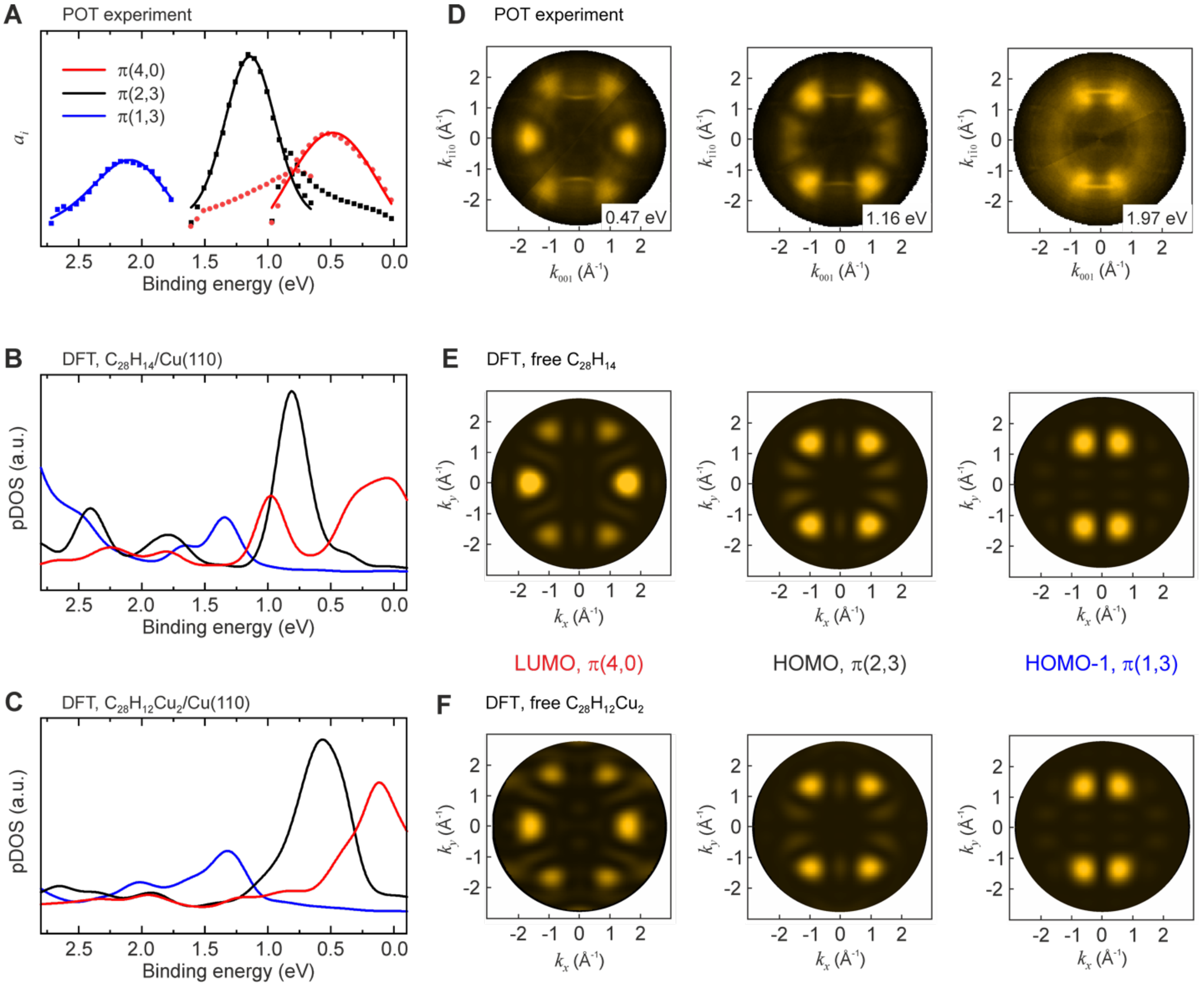

Fig. S1. Frontier π-orbitals of two bisanthene-like species on Cu(110). (**A**) Experimental orbital projected density of states (pDOS) obtained by deconvolution of the photoemission orbital tomography (POT) results using the theoretical $\mathbf{k}_{\parallel}$-maps of free bisanthene ($C_{28}H_{14}$, **4**) shown in panel **E**. Note that deconvolution using the theoretical $\mathbf{k}_{\parallel}$-maps of free metalated bisanthene ($C_{28}H_{12}Cu_2$, **5**) shown in panel **F** delivers qualitatively similar results. (**B**) Theoretical pDOS of $C_{28}H_{14}$/Cu(110) calculated by van-der-Waals-corrected DFT with the PBE-GGA functional. (**C**) Theoretical pDOS of $C_{28}H_{12}Cu_2$/Cu(110) calculated by van-der-Waals-corrected DFT with the PBE-GGA functional. (**D**) Experimental $\mathbf{k}_{\parallel}$-maps recorded at different binding energies E_b. (**E**) Theoretical $\mathbf{k}_{\parallel}$-maps calculated for LUMO, HOMO and HOMO-1 of free bisanthene ($C_{28}H_{14}$, **4**). (**F**) Theoretical $\mathbf{k}_{\parallel}$-maps calculated for LUMO, HOMO and HOMO-1 of free metalated bisanthene ($C_{28}H_{12}Cu_2$, **5**).

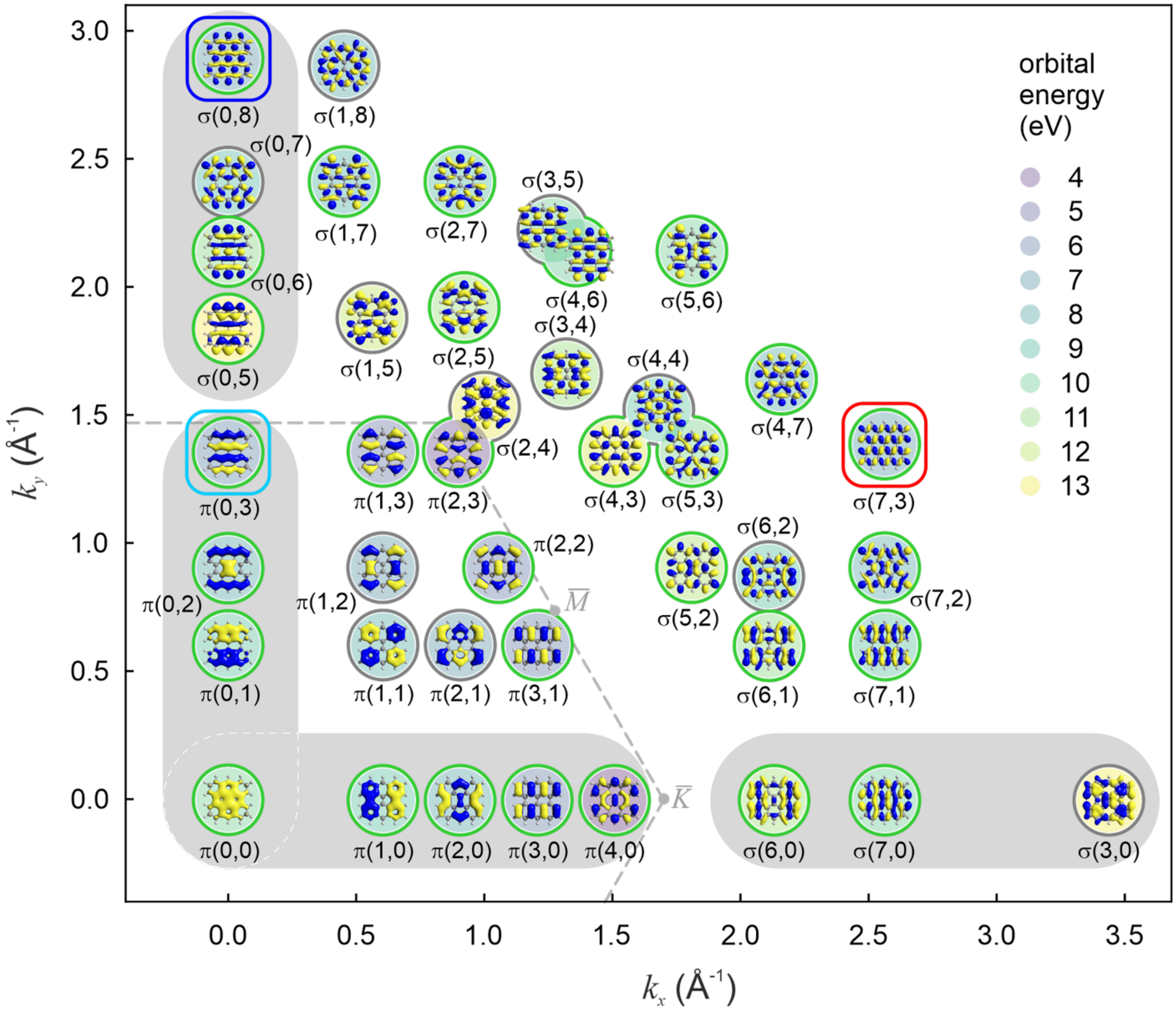

Fig. S2. Orbitals of bisanthene ($C_{28}H_{14}$, 4). The DFT-calculated theoretical orbitals of the free molecule are arranged according to the (k_x, k_y)-positions of their smallest $|\mathbf{k}_\parallel|$ emission lobes in their respective $\mathbf{k}_\parallel$-maps. The orbitals are labeled according to the number of nodal planes along the two principal directions (cf. main text). Calculated orbital energies with respect to the vacuum level are indicated by the color scale. Circles around the orbitals indicate whether they have been identified (green) or not identified (gray) in the experimental dataset. Red, blue and light blue frames mark the σ(7,3), σ(0,8) and π(0,3) orbitals, respectively. The gray shaded areas denote orbitals of the π and σ-bands marked in Fig. 2**B**,**C**. As a reference length scale in momentum space, the dashed gray line marks the Brillouin zone of graphene.

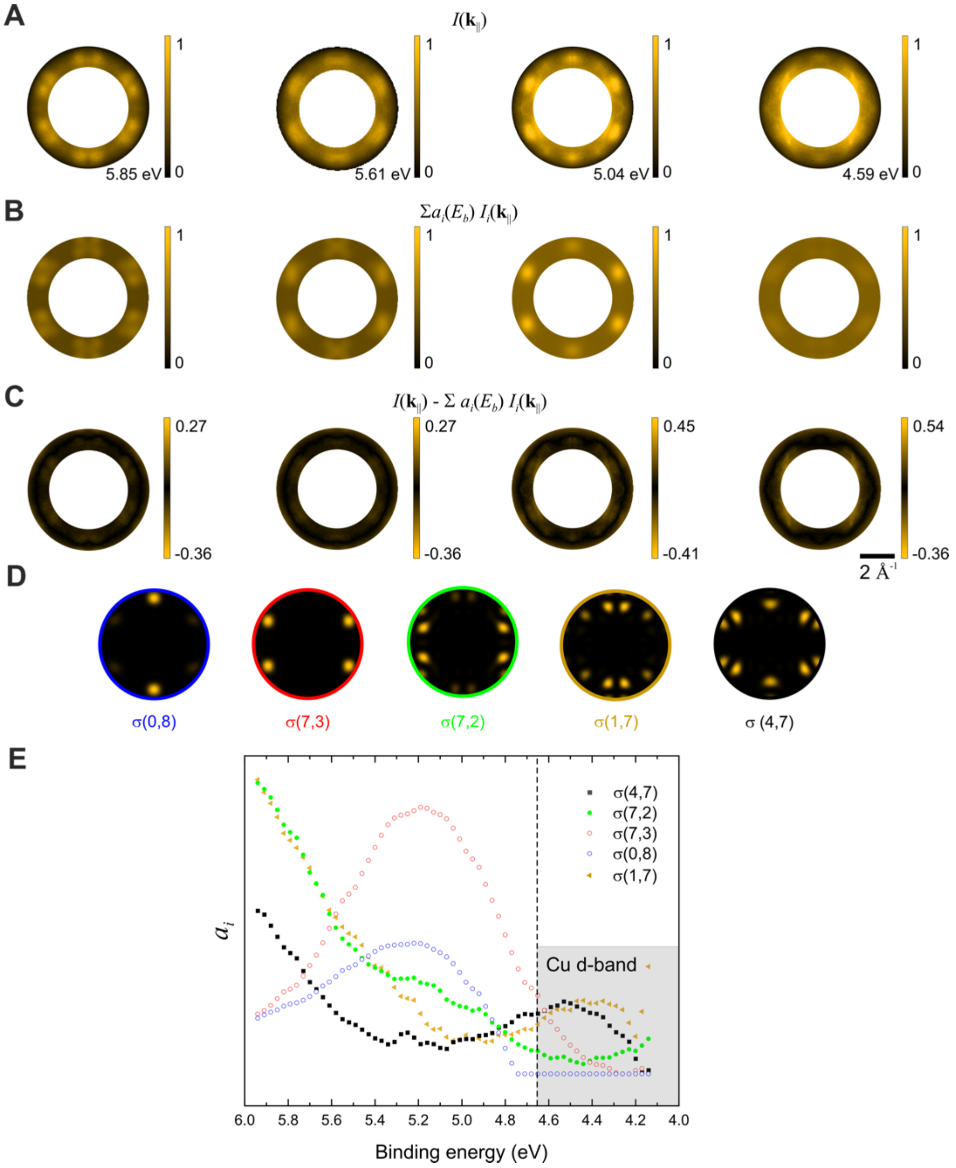

Fig. S3. Deconvolution of the experimental POT results in the E_b range from 4.15 to 6.00 eV. (**A**) Experimental $\mathbf{k}_\parallel$-maps $I(\mathbf{k}_\parallel)$, corresponding binding energies are given in the lower right corners of the maps. (**B**) $\mathbf{k}_\parallel$-maps $\sum_i a_i\,(E_b) I_i(\mathbf{k}_\parallel)$ constructed as intensity sum of theoretical $\mathbf{k}_\parallel$-maps $I_i(\mathbf{k}_\parallel)$ of free bisanthene ($C_{28}H_{14}$, **4**) shown in panel **D** and weighted using the fitted parameters $a_i(E_b)$ from panel **E**. (**C**) Deconvolution residual $I(\mathbf{k}_\parallel)$- $\sum_i a_i\,(E_b) I_i(\mathbf{k}_\parallel)$. (**D**) Theoretical $\mathbf{k}_\parallel$-maps $I_i(\mathbf{k}_\parallel)$ of the σ-orbitals of free bisanthene ($C_{28}H_{14}$, **4**) used for deconvolution. (**E**) Fitting parameters $a_i(E_b)$ – the experimental pDOS of the σ-orbitals obtained from deconvolution. All $\mathbf{k}_\parallel$-maps are restricted to $|\mathbf{k}_\parallel| \geq 2.2$ Å^{-1} to exclude low-$|\mathbf{k}_\parallel|$ emissions from π-states.

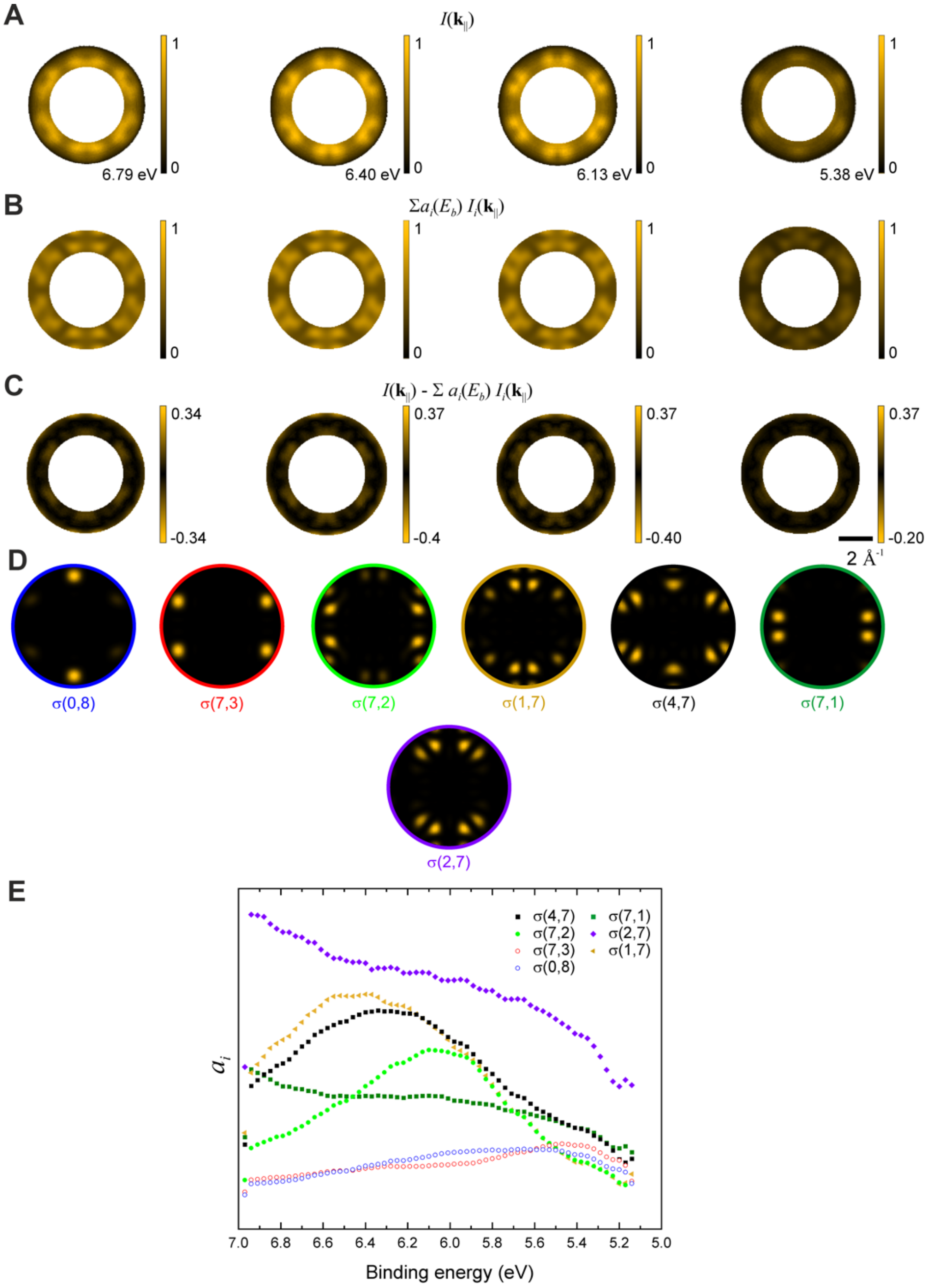

Fig. S4. Deconvolution of the experimental POT results in the E_b range from 5.15 to 7.00 eV. (**A**) Experimental $\mathbf{k}_\parallel$-maps $I(\mathbf{k}_\parallel)$, corresponding binding energies are given in the lower right corners of the maps. (**B**) $\mathbf{k}_\parallel$-maps $\sum_i a_i\,(E_b) I_i(\mathbf{k}_\parallel)$ constructed as intensity sum of theoretical $\mathbf{k}_\parallel$-maps $I_i(\mathbf{k}_\parallel)$ of free bisanthene ($C_{28}H_{14}$, **4**) shown in panel **D** and weighted using the fitted parameters $a_i(E_b)$ from panel **E**. (**C**) Deconvolution residual $I(\mathbf{k}_\parallel)$- $\sum_i a_i\,(E_b) I_i(\mathbf{k}_\parallel)$. (**D**) Theoretical $\mathbf{k}_\parallel$-maps $I_i(\mathbf{k}_\parallel)$ of the σ-orbitals of free bisanthene ($C_{28}H_{14}$, **4**) used for deconvolution. (**E**) Fitting parameters $a_i(E_b)$ – the experimental pDOS of the σ-orbitals obtained from deconvolution. All $\mathbf{k}_\parallel$-maps are restricted to $|\mathbf{k}_\parallel| \geq 2.2$ Å^{-1} to exclude low-$|\mathbf{k}_\parallel|$ emissions from π-states.

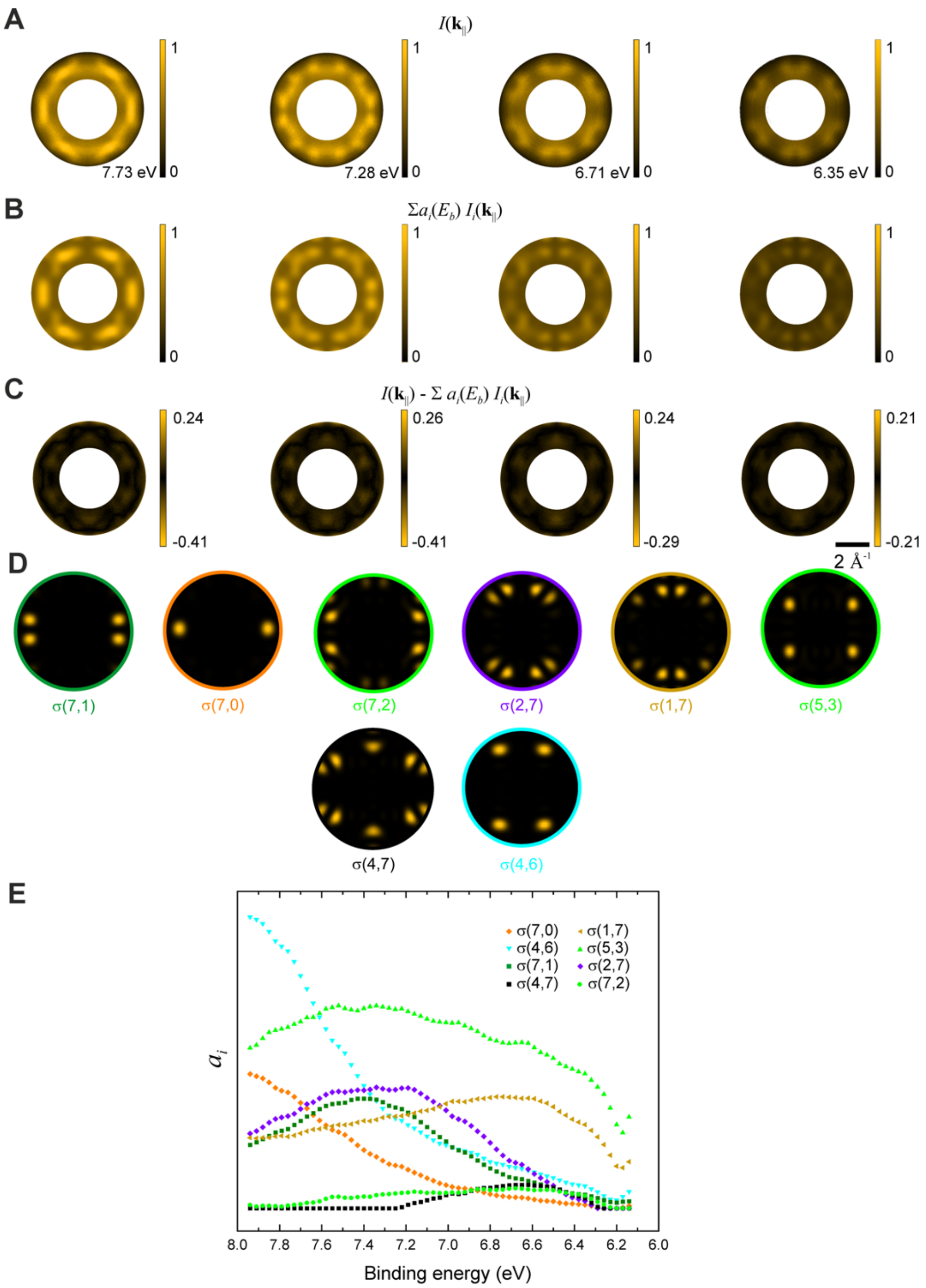

Fig. S5. Deconvolution of the experimental POT results in the E_b range from 6.15 to 8.00 eV. (**A**) Experimental $\mathbf{k}_{\|}$-maps $I(\mathbf{k}_{\|})$, corresponding binding energies are given in the lower right corners of the maps. (**B**) $\mathbf{k}_{\|}$-maps $\sum_i a_i\,(E_b) I_i(\mathbf{k}_{\|})$ constructed as intensity sum of theoretical $\mathbf{k}_{\|}$-maps $I_i(\mathbf{k}_{\|})$ of free bisanthene ($C_{28}H_{14}$, **4**) shown in panel **D** and weighted using the fitted parameters $a_i(E_b)$ from panel **E**. (**C**) Deconvolution residual $I(\mathbf{k}_{\|})$- $\sum_i a_i\,(E_b) I_i(\mathbf{k}_{\|})$. (**D**) Theoretical $\mathbf{k}_{\|}$-maps $I_i(\mathbf{k}_{\|})$ of the σ-orbitals of free bisanthene ($C_{28}H_{14}$, **4**) used for deconvolution. (**E**) Fitting parameters $a_i(E_b)$ – the experimental pDOS of the σ-orbitals obtained from deconvolution. All $\mathbf{k}_{\|}$-maps are restricted to $|\mathbf{k}_{\|}| \geq 1.6$ Å^{-1} to exclude low-$|\mathbf{k}_{\|}|$ emissions from π-states.

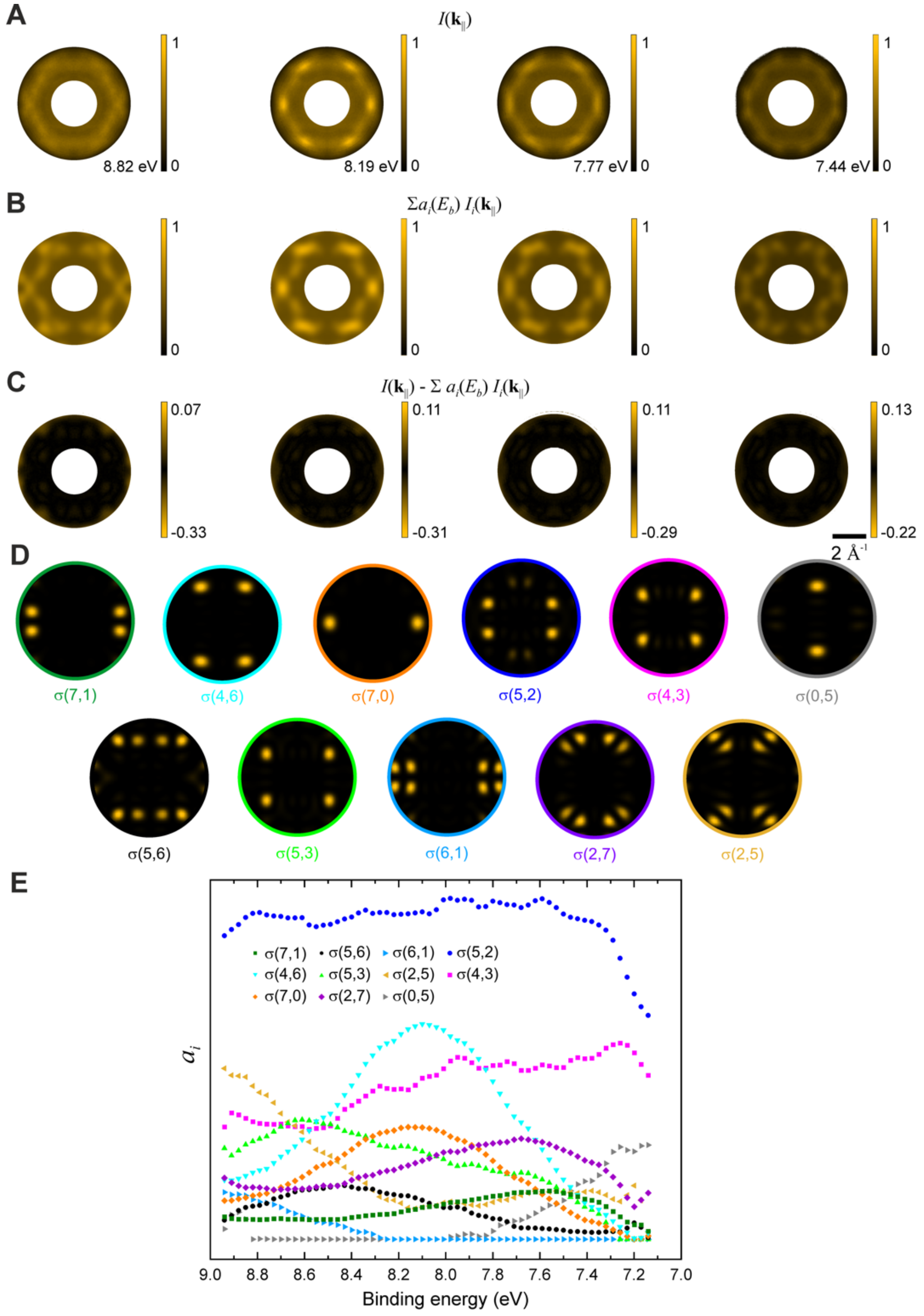

Fig. S6. Deconvolution of the experimental POT results in the E_b range from 7.15 to 9.00 eV. (**A**) Experimental $\mathbf{k}_{\|}$-maps $I(\mathbf{k}_{\|})$, corresponding binding energies are given in the lower right corners of the maps. (**B**) $\mathbf{k}_{\|}$-maps $\sum_i a_i(E_b) I_i(\mathbf{k}_{\|})$ constructed as intensity sum of theoretical $\mathbf{k}_{\|}$-maps $I_i(\mathbf{k}_{\|})$ of free bisanthene ($C_{28}H_{14}$, **4**) shown in panel **D** and weighted using the fitted parameters $a_i(E_b)$ from panel **E**. (**C**) Deconvolution residual $I(\mathbf{k}_{\|})$- $\sum_i a_i(E_b) I_i(\mathbf{k}_{\|})$. (**D**) Theoretical $\mathbf{k}_{\|}$-maps $I_i(\mathbf{k}_{\|})$ of the σ-orbitals of free bisanthene ($C_{28}H_{14}$, **4**) used for deconvolution. (**E**) Fitting parameters $a_i(E_b)$ – the experimental pDOS of the σ-orbitals obtained from deconvolution. All $\mathbf{k}_{\|}$-maps are restricted to $|\mathbf{k}_{\|}| \geq 1.3$ Å^{-1} to exclude low-$|\mathbf{k}_{\|}|$ emissions from π-states.

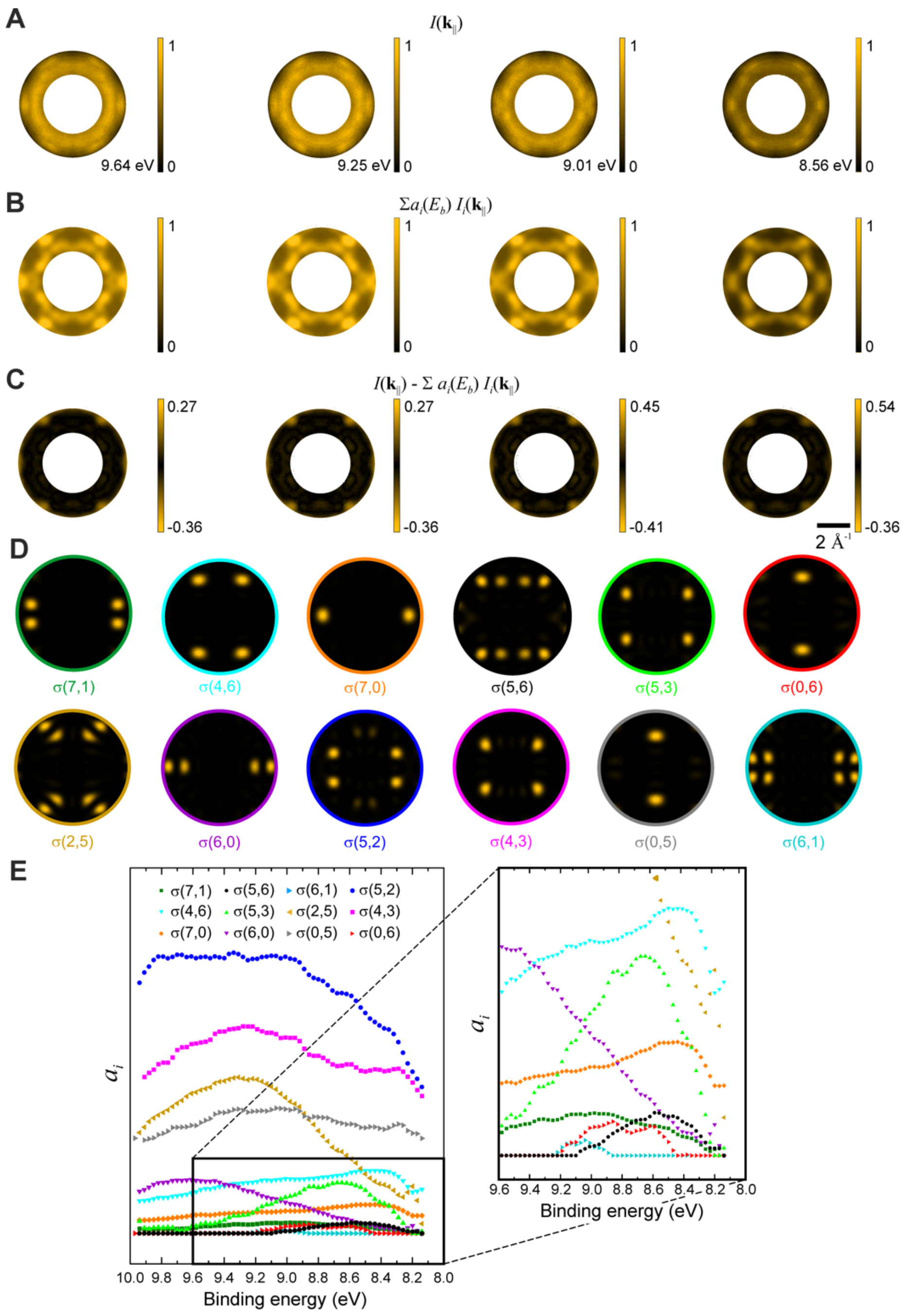

Fig. S7. Deconvolution of the experimental POT results in the E_b range from 8.15 to 10.00 eV. (**A**) Experimental $\mathbf{k}_{\parallel}$-maps $I(\mathbf{k}_{\parallel})$, corresponding binding energies are given in the lower right corners of the maps. (**B**) $\mathbf{k}_{\parallel}$-maps $\sum_i a_i\,(E_b) I_i(\mathbf{k}_{\parallel})$ constructed as intensity sum of theoretical $\mathbf{k}_{\parallel}$-maps $I_i(\mathbf{k}_{\parallel})$ of free bisanthene ($C_{28}H_{14}$, **4**) shown in panel **D** and weighted using the fitted parameters $a_i(E_b)$ from panel **E**. (**C**) Deconvolution residual $I(\mathbf{k}_{\parallel})$- $\sum_i a_i\,(E_b) I_i(\mathbf{k}_{\parallel})$. (**D**) Theoretical $\mathbf{k}_{\parallel}$-maps $I_i(\mathbf{k}_{\parallel})$ of the σ-orbitals of free bisanthene ($C_{28}H_{14}$, **4**) used for deconvolution. (**E**) Fitting parameters $a_i(E_b)$ – the experimental pDOS of the σ-orbitals obtained from deconvolution. All $\mathbf{k}_{\parallel}$-maps are restricted to $|\mathbf{k}_{\parallel}| \geq 1.7$ Å^{-1} to exclude low-$|\mathbf{k}_{\parallel}|$ emissions from π-states.

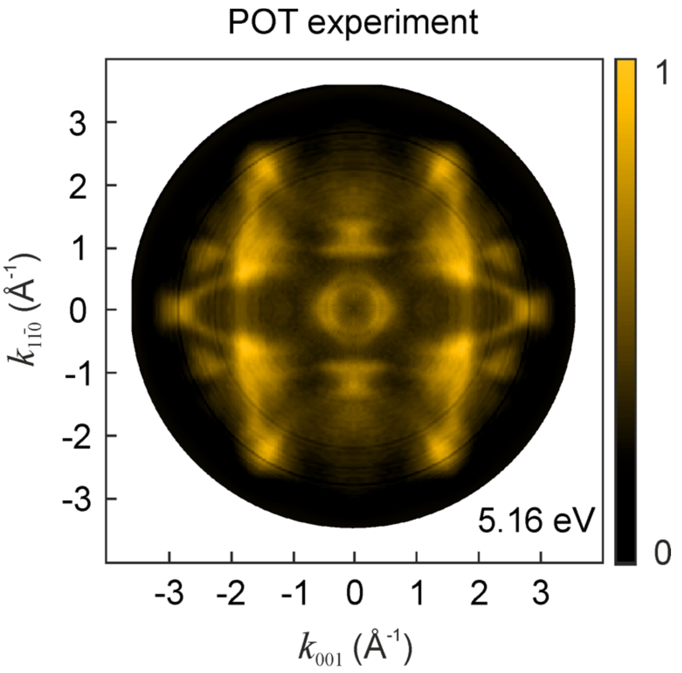

Fig. S8. Experimental k$_\parallel$-map of clean Cu(110). Experimental **k**$_\parallel$-map recorded at E_b = 5.16 eV and photon energy $h\nu$ = 57 eV. A uniform background has been subtracted from the map.

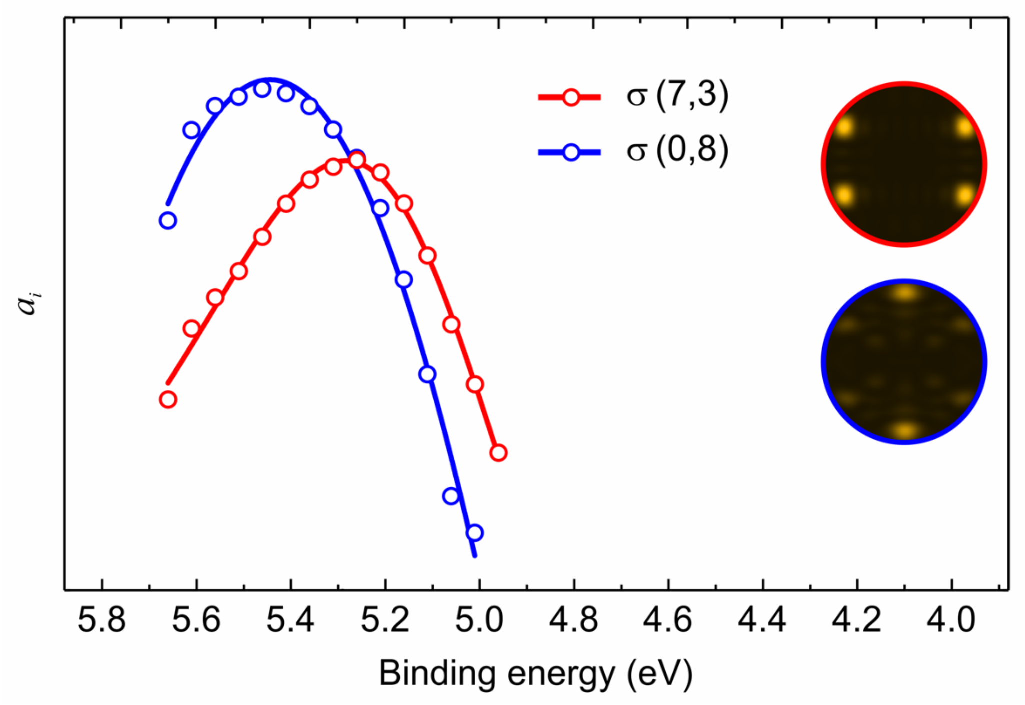

Fig. S9. Deconvolution of experimental POT results. Experimental pDOS of σ(7,3) (red) and σ(0,8) (blue) orbitals. The data points were obtained from the deconvolution of the experimental $I(E_b, \mathbf{k}_\parallel)$ data cube, using the theoretical $\mathbf{k}_\parallel$-maps of free metalated bisanthene ($C_{28}H_{12}Cu_2$, **5**) shown in the inset. Solid lines are Gaussian fits to the data points.

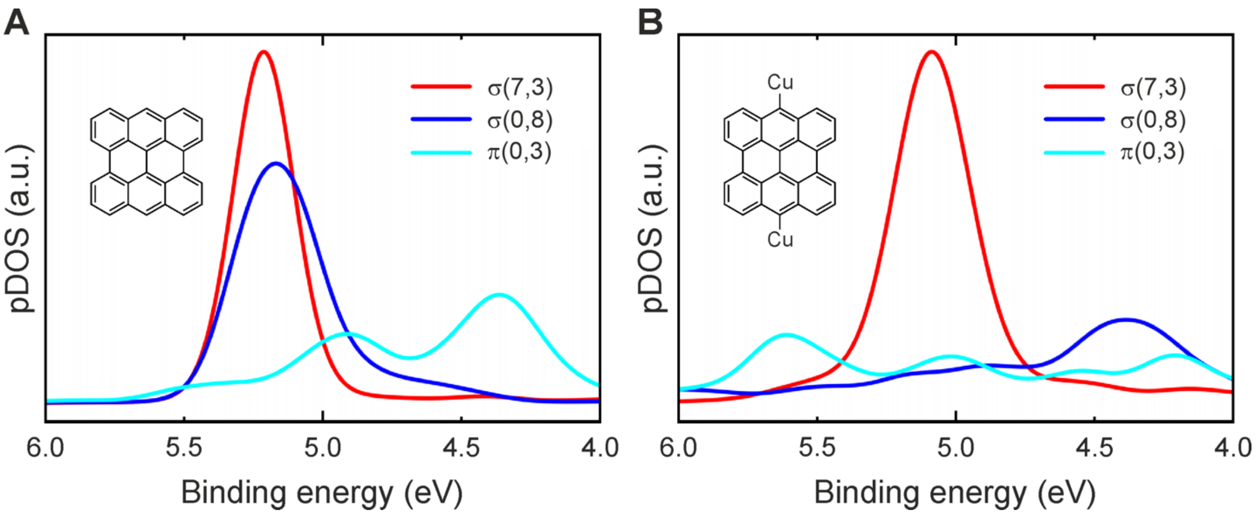

Fig. S10. Theoretical pDOS of two bisanthene-like species on Cu(110). Molecular orbital pDOS based on van-der-Waals-corrected DFT calculations with the hybrid functional HSE06 for (**A**) $C_{28}H_{14}$/Cu(110) and (**B**) $C_{28}H_{12}Cu_2$/Cu(110). Projections onto three orbitals σ(7,3), σ(0,8) and π(0,3) are shown.

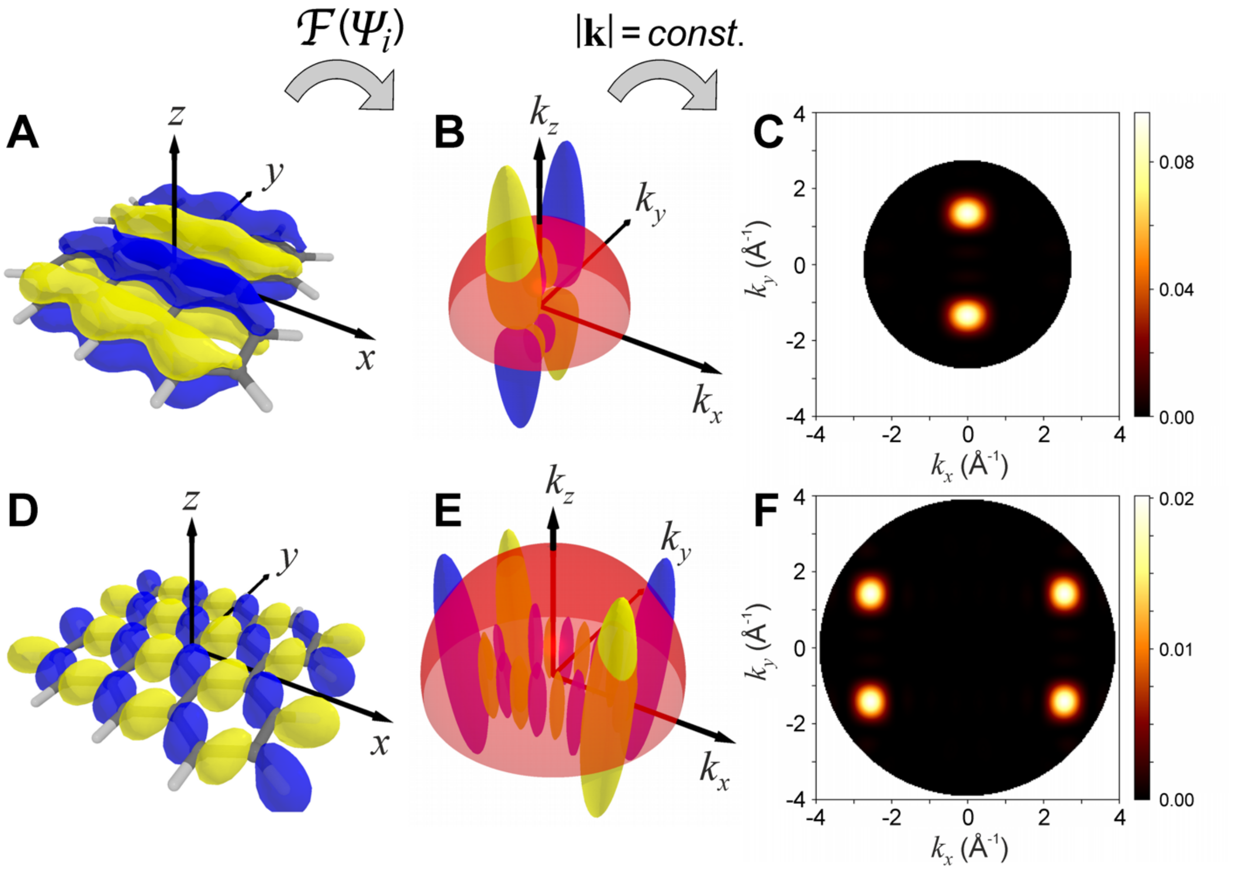

Fig. S11. Illustration of the generation of theoretical photoemission $\mathbf{k}_{\parallel}$-maps from gas-phase orbitals. The procedure is exemplified for the bisanthene ($C_{28}H_{14}$, **4**) orbitals (**A**) π(0,3) and (**D**) σ(7,3). Starting from the orbitals in real space (**A**,**D**) $\Psi(x, y, z)$, a three-dimensional Fourier transform leads to the momentum-space representation of the orbitals (**B**,**E**) $\widetilde{\Psi}(k_x, k_y, k_z)$. According to Eq. 5, a hemispherical cut (red) at $|\mathbf{k}| = \sqrt{2mE_{kin}/\hbar^2}$ multiplied with the polarization factor then leads to the photoemission intensity $I_i(k_x, k_y)$, the $\mathbf{k}_{\parallel}$-map (**C**,**F**). Kinetic energies E_{kin} of 30 and 60 eV have been used in this illustration for π(0,3) and σ(7,3), respectively. Note a perspective projection used in panels **A**,**B**,**D**,**E**.

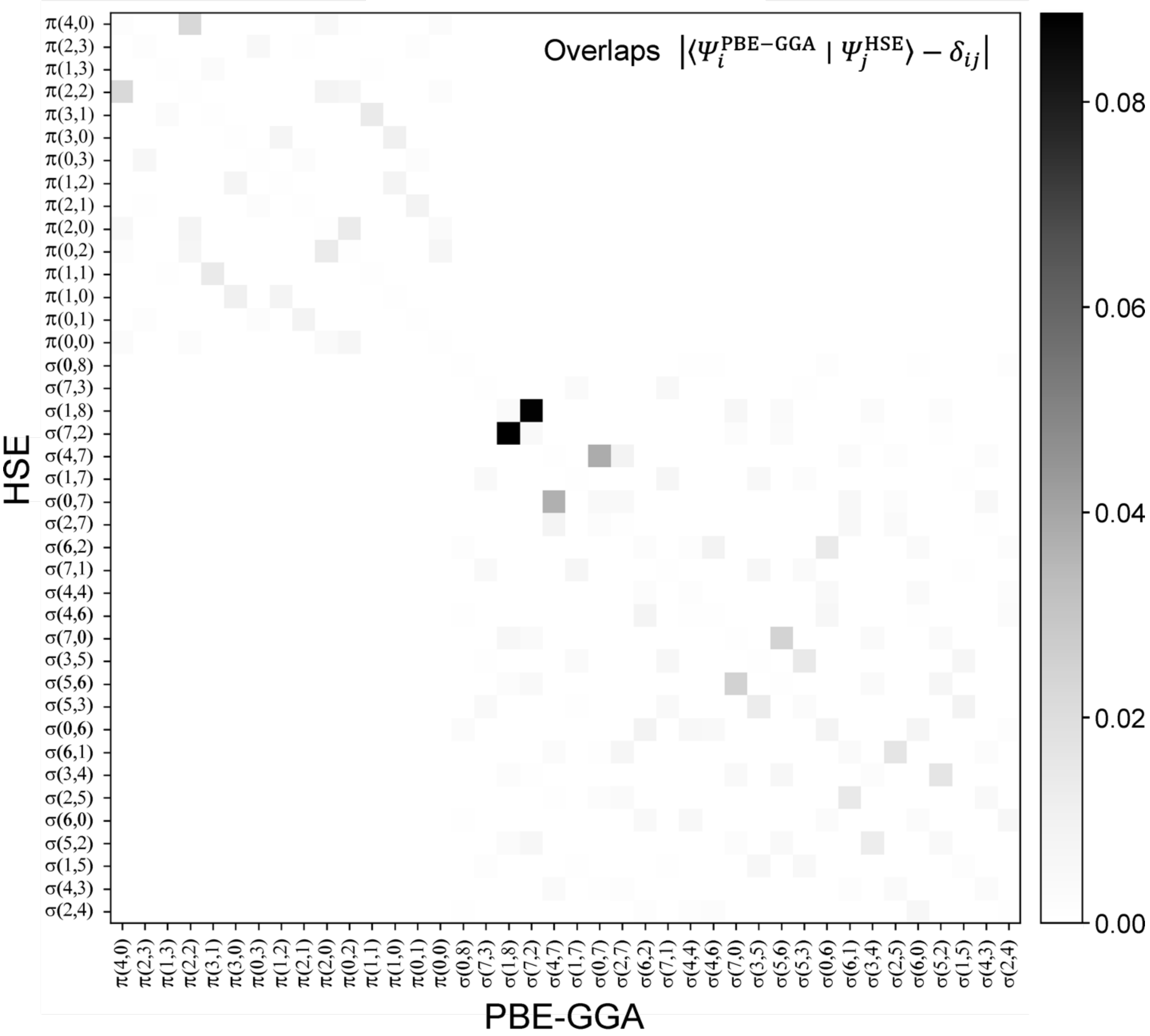

Fig. S12. PBE-GGA vs HSE. Comparison of the orbitals of free bisanthene ($C_{28}H_{14}$, **4**) computed with the PBE-GGA functional (horizontal axis) and the range-separated hybrid HSE06 functional (vertical axis). The overlap of these two sets of orbitals compared to the identity matrix are plotted as a gray-scale density plot. See Eq. 7 for the mathematical definition of the displayed quantity.

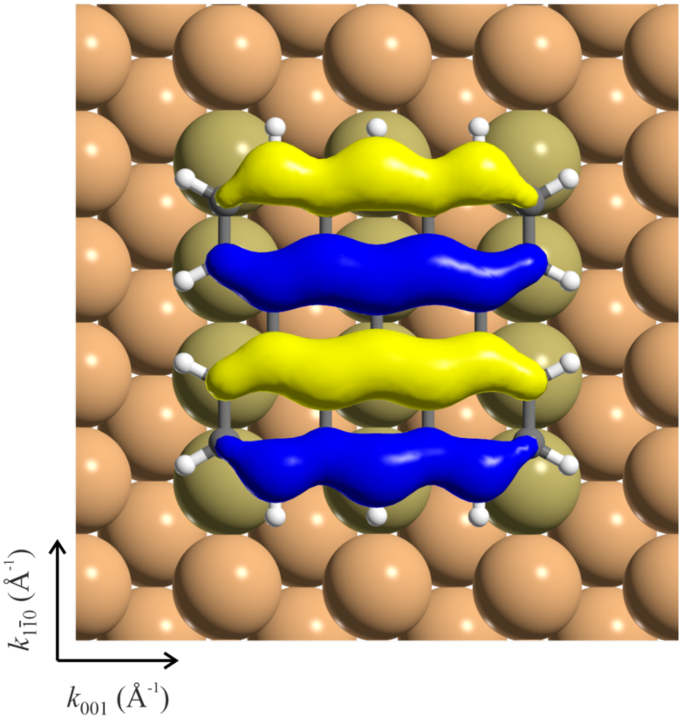

Fig. S13. Bisanthene on Cu(110). The energetically favored adsorption site of bisanthene ($C_{28}H_{14}$, **4**) on Cu(110) according to van-der-Waals-corrected DFT using the PBE-GGA functional. The π(0,3) orbital calculated by DFT for free $C_{28}H_{14}$ is superimposed with the molecular backbone.

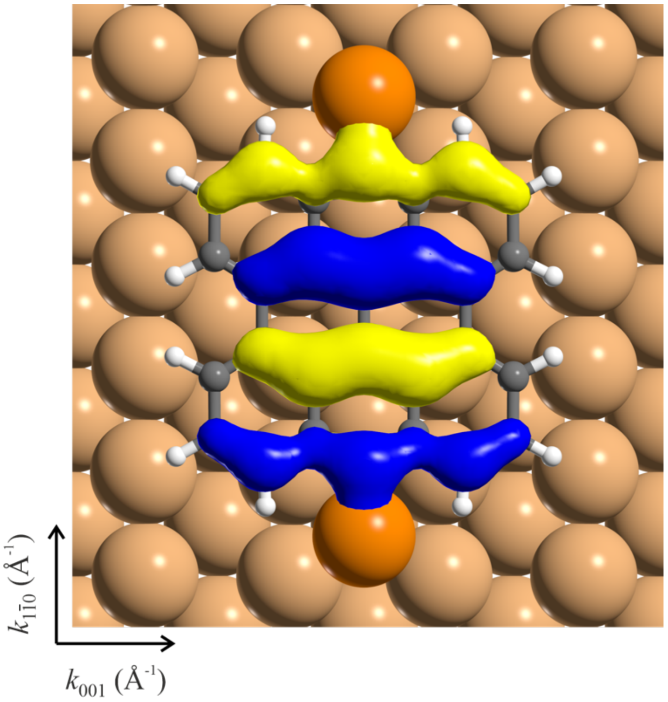

Fig. S14. Metalated bisanthene on Cu(110). The energetically favored adsorption site of metalated bisanthene ($C_{28}H_{12}Cu_2$, **5**) on Cu(110) according to van-der-Waals-corrected DFT using the PBE-GGA functional. The π(0,3) orbital calculated by DFT for free $C_{28}H_{12}Cu_2$ is superimposed with the molecular backbone.

Movie S1. Experimental $I(E_b, \mathbf{k}_\parallel)$ data cube of POT. Animated representation of experimental data (photoemission intensity distribution as a function of $\mathbf{k}_\parallel$ and binding energy E_b), measured at photon energy $h\nu = 57$ eV.
